# Parvovirus B19 Replication and Expression in Differentiating Erythroid Progenitor Cells

**DOI:** 10.1371/journal.pone.0148547

**Published:** 2016-02-04

**Authors:** Gloria Bua, Elisabetta Manaresi, Francesca Bonvicini, Giorgio Gallinella

**Affiliations:** 1 Department of Pharmacy and Biotechnology, University of Bologna, Bologna, Italy; 2 S.Orsola-Malpighi Hospital – Microbiology, University of Bologna, Bologna, Italy; University of Kansas Medical Center, UNITED STATES

## Abstract

The pathogenic Parvovirus B19 (B19V) is characterized by a strict adaptation to erythroid progenitor cells (EPCs), a heterogeneous population of differentiating cells with diverse phenotypic and functional properties. In our work, we studied the dynamics of B19V infection in EPCs in dependence on the cell differentiation stage, in terms of distribution of infected cells, synthesis of viral nucleic acids and production of infectious virus. EPCs at early differentiation stage led to an abortive infection, without viral genome replication and a very low transcriptional activity. EPCs at later stages were permissive, with highest levels of viral replicative activity at day 9 (+3.0 Log from 2 to 48 hpi) and lower levels at day 18 (+1.5 Log from 2 to 48 hpi). B19V DNA increment was in accordance with the percentage of cells positive to flow-FISH assay (41.4% at day 9, 1.1% at day 18). Quantitation of total RNA indicated a close association of genome replication and transcription with viral RNA accumulation within infected cells related to viral DNA increase during the course of infection. Analysis of the different classes of mRNAs revealed two distinct pattern of genome expression profile with a fine regulation in the frequency utilization of RNA processing signals: an early phase, when cleavage at the proximal site leading to a higher relative production of mRNA for NS protein, and a late phase, when cleavage at the distal site was more frequent leading to higher relative abundance of mRNA for VP and 11 kDA proteins. Infectious virus was released from cells at day 6–15, but not at day 18. Our results, providing a detailed description of B19V replication and expression profile in differentiating EPCs, highlight the very tight adaptation of B19V to a specific cellular target defined both by its erythroid lineage and its differentiation stage.

## Introduction

In the Parvoviridae family [1], Parvovirus B19 (B19V) is a human virus with an ample pathogenic potential [2,3]. B19V has a selective tropism for the erythroid lineage in the bone marrow, where productive infection induces a block in erythropoiesis that can be manifested as a transient or persistent erythroid aplasia [4]. Anemia as a consequence of the block in erythropoiesis usually becomes clinically relevant when an underlying condition is present, such as expanded erythropoiesis compensating for hematological disorders, or in case of inadequacy of the specific antiviral immune response [5]. Apart from hematological consequences, B19V commonly causes erythema infectiosum in children, arthropathies, mainly affecting adults, and it is implicated in a growing spectrum of diverse pathologies and inflammatory processes affecting various tissues and organs [6]. During pregnancy, the tropism of B19V for erythroid progenitors in liver and bone marrow can lead to fetal anemia, tissue hypoxia, development of non-immune hydrops and/or fetal death [7–9].

The main target for B19V replication, the erythroid compartment in the bone marrow, is a heterogeneous population of proliferating and differentiating cells, a composite set with different phenotypic and functional aspects that are likely to affect the properties and outcome of infection [10–12]. As an experimental system, an expanding population of erythroid progenitor cells (EPCs) can be obtained from peripheral blood mononuclear cells (PBMC), by culturing in medium enriched with appropriate growth and differentiating factors. Such cellular system replicates in vitro the differentiation process that occurs in vivo in the bone marrow environment, thus consenting the study of the changes in cellular expression patterns that occur during erythroid differentiation in the different cell subsets. Notably, this cellular system has been shown to support B19V replication to significant extents [13,14].

Investigation of B19V-cell interactions in PBMC-derived EPCs may permit a thorough description of events related to the viral replication cycle, reproducing the characteristics of the corresponding cells in bone marrow more appropriately than cell lines models such as UT7/EpoS1 or analogous, where some degrees of restriction to viral replication, related to different mechanisms, invariably occur [15,16]. Furthermore, the same heterogeneity of the EPCs population in terms of proliferating and differentiating status constitutes a challenging system to investigate the dependence of viral replication on cell characteristics. In our study, we used PBMC derived EPCs, at different days of in vitro culture corresponding to different phases of erythroid differentiation, as a target cell population to define the dynamics of B19V infection, in terms of distribution of productively infected cells, dynamics of viral nucleic acids macromolecular synthesis, and production of infectious virus. The results led to the definition of a model of B19V replication in EPCs, in dependence on the cell population differentiation stage.

## Materials and Methods

### Cells

Erythroid progenitor cells (EPCs) were generated in vitro from peripheral blood mononuclear cells (PBMC) obtained from the leukocyte-enriched buffy coats of anonymous blood donors, available for institutional research purposes from the Immunohematology and Transfusion Service, S.Orsola-Malpighi University Hospital, Bologna (http://www.aosp.bo.it/content/immunoematologia-e-trasfusionale; authorization 0070755/1980/2014, issued by Head of Service). Availability was granted under conditions complying with Italian privacy law. Neither specific ethics committee approval nor written consent from donors was required for this research project. PBMC were isolated using centrifugation in Ficoll-Paque Plus (GE Healthcare Bio-Sciences AB) and cultured in a medium containing erythropoietic growth and differentiation factors, following previously established protocols with minor modifications [14].

Isolated PBMC were cultured in IMDM supplemented with the serum substitute BIT 9500 (StemCell Technologies), diluted 1:5 for a final concentration of 10 mg/ml bovine serum albumin, 10 mg/ml recombinant human (rhu) insulin, and 200 mg/ml iron-saturated human transferrin, enriched with 900 ng/ml ferrous sulphate, 90 ng/ml ferric nitrate, 1 mM hydrocortisone (Sigma), 3 IU/ml rhu erythropoietin (NeoRecormon, Roche), 5 ng/ml rhu IL-3 and 20 ng/ml rhu stem cell factor (Life Technologies,). The cells were maintained at 37°C in 5% CO_2_, at a density of 0.5/1.0x10^6^ cells/mL for up to 20 days post-isolation.

### Flow Cytometry Analysis

In vitro cultured EPCs were characterized using flow cytometry (FACSCalibur, Becton Dickinson). Aliquots of 5 x 10^5^ EPCs were stained with antibodies specific for erythroid differentiation markers (CD36, CD71, CD235a) and known B19V receptors (globoside, α5β1 integrin). CD36 and CD71 expression was evaluated by phycoerythrin (PE)-labeled monoclonal antibodies (BD Biosciences). CD235a, α5β1 integrin and globoside expression was evaluated by monoclonal mouse anti-CD235a (BD Biosciences), monoclonal mouse anti- α5β1 (Immunological Sciences) and polyclonal rabbit anti-globoside (Matreya), followed by anti-mouse-Alexa 488 (Life Technologies) or anti-rabbit- FITC (DakoCytomation) antibody, respectively. Data were analyzed using the Cell Quest Pro Software (Becton Dickinson).

### Virus and Infection

A B19V viremic serum sample, identified in our laboratory in the course of routine diagnostic analysis and available for research purposes according to Italian privacy law, was used as source of virus for the infection experiments. The viremic serum contained 10^12^ B19V (genotype 1) genome copies (geq)/mL, as determined by quantitative PCR analysis, and resulted negative by routine diagnostic assays to other viruses [17].

For infection of EPCs, cells at the density of 10^7^ cells/mL were incubated in the presence of 0.1x volume of viremic serum, appropriately diluted in PBS in order to obtain a multiplicity of infection (moi) of 10^3^ geq/cell. Following adsorption for 2 h at 37°C, the inoculum virus was washed and the cells were incubated at 37°C in complete medium at an initial density of 5x10^5^ cells/ml, for a time course of infection.

### Flow-FISH Assay

Constant volumes of cell cultures, containing about 1x10^6^ cells, were collected at the appropriate time points post-infection and processed by flow-FISH assay for the detection of viral nucleic acids, as described [18]. Cells were fixed in PBS-paraformaldehyde 0.5% at 4°C overnight, permeabilized by resuspending in PBS containing 0.2% saponin, and finally resuspended in 50 μl of a hybridization solution containing 70% formamide. A digoxigenin-labeled DNA probe mixture specific for B19V nucleic acids was generated by the random priming method on a cloned full-length genomic DNA template, according to the manufacturer’s instructions (Dig-High Prime, Roche). The probe mixture was separately denatured at 95°C for 5min, then added at 2 μg/mL to the cell suspension, previously heated at 70°C for 5 min. Hybridization occurred at 37°C for 12 h, then cells were washed at RT for 15 min in 1x Stringent Wash (Zytovision). For the detection of the hybrids, the cell suspension was incubated for 1 h with a FITC-conjugate antidigoxigenin antibody diluted 1:20 in PBS-BSA 1%, washed twice in PBS and re-suspended in 500 μl PBS for subsequent flow cytometry analysis. Mock-infected cells were treated equally and used for gating; 50000 events were counted for each sample analyzed. To discriminate B19V infected cells, for each experimental series and for both infected and mock-infected cells, a fixed gate was set on the FSC versus FITC dot plot in order to maintain at least 99.5% of mock-infected cells below threshold. Cells showing fluorescence intensities greater than the negative gate were considered positive for B19V nucleic acids, and the neat percentage of B19V positive cells was evaluated by determining the number of cells above threshold in the infected cell samples and subtracting the number of cells above threshold in the respective negative controls.

### Nucleic acids purification

Equal amounts of cell cultures, corresponding to 2x10^5^ cells, were collected at the appropriate time points following infection (hours post-infection, hpi) and processed by using the EZ1 viral nucleic acid kit on a EZ1 platform (Qiagen), following the manufacturer’s instructions, in order to obtain a total nucleic acid fraction, containing both viral DNA and RNA in elution volumes of 120 μL. Volumes of 10 μL were then used in the subsequent qPCR and qRT-PCR assays for the quantitative evaluation of target viral nucleic acids. For quantitative analysis of viral DNA, aliquots of the eluted nucleic acids were directly amplified in a qPCR assay. For quantitative analysis of viral RNA, corresponding aliquots were first treated with Turbo DNAfree reagent (Ambion) and then amplified in a qRT-PCR assay.

### Quantitative real-time PCR and RT-PCR

**Fig 1:**
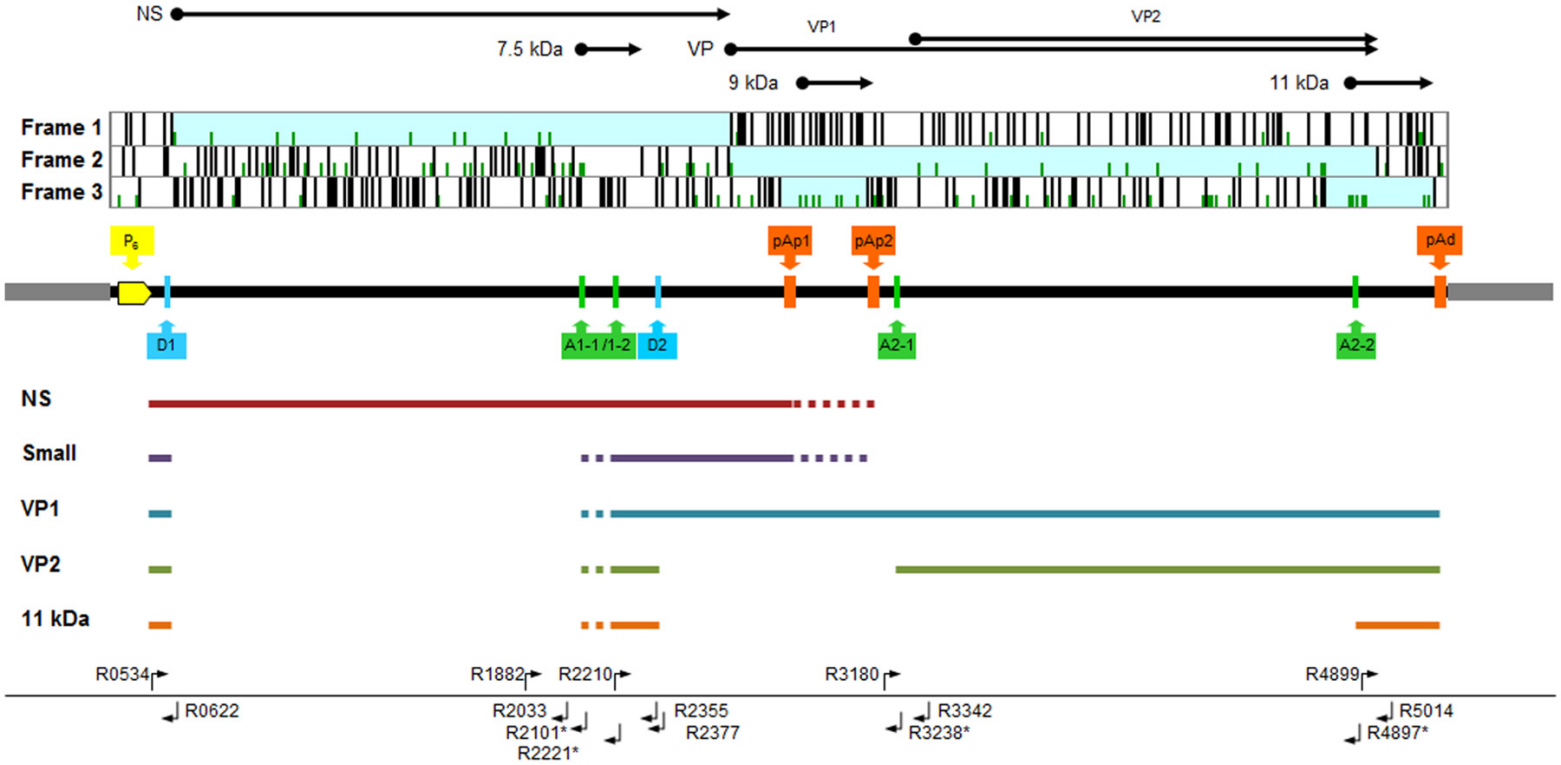
Outline of parvovirus B19 genome structure and organization. Top, ORF distribution within B19V internal coding region and related coding sequences. Center, functional map of B19V genome and distribution of regulatory signals: grey boxes, inverted terminal regions; P_6_, promoter region; pAp1, pAp2, pAd: proximal and distal cleavage-polyadenylation sites; D1, A1-1/2, D2, A2-1/2, donor and alternative acceptor sites for introns 1 and 2, respectively. Below, a simplified map of B19V transcripts. Bottom, position of primers used in the PCR array (Table 1).

Standard targets for the amplification reactions were obtained from plasmid pHR0, that contains an insert corresponding to the complete internal region of B19V genome (nt. 346–5245) [19]. From pHR0 plasmid, in vitro amplified DNA or in vitro transcribed RNA corresponding to the viral insert were obtained, purified, quantified and serially diluted to obtain calibration standards. Primers and primer combinations used in the qPCR and qRT-PCR assays are indicated in Table 1 and located with respect to the functional map of B19V genome in Fig 1 [3,20,21]. All oligonucleotides were obtained from MWG Biotech.

**Table 1:** Primers used in the qPCR and qRT-PCR assays for the detection and quantitative evaluation of B19V nucleic acids.

**Primer**	**Sense**	**Primer**	**Antisense**	**DNA Target**
18Sfor	CGGACAGGATTGACAGATTG	18Srev	TGCCAGAGTCTCGTTCGTTA	Genomic 18S rDNA
R2210	CGCCTGGAACACTGAAACCC	R2355	GAAACTGGTCTGCCAAAGGT	Virus DNA
**Primer**	**Sense**	**Primer**	**Antisense**	**RNA Target**
R0534	TGGGCTGCTTTTTCCTGGAC	R0622	ATAGCTCCATGTTAGTATGT	Intron 1, unspliced
		R2101*	CTGGGTGGAGGGCATCTGTT	Intron 1, splice D1/A1.1
		R2221*	CAGTGTTCCAGGCGCCTGTT	Intron 1, splice D1/A1.2
R1882	GCGGGAACACTACAACAACT	R2033	GTCCCAGCTTTGTGCATTAC	Intron 1, unspliced
R2210	CGCCTGGAACACTGAAACCC	R2355	GAAACTGGTCTGCCAAAGGT	Central exon, total RNA
R2210	CGCCTGGAACACTGAAACCC	R2377	TCAACCCCAACTAACAGTTC	Intron 2, unspliced
		R3238*	CAGGGGCAGCTGCACAGTTC	Intron 2, splice D2/A2.1
		R4897*	GTTTTGCATCTGTAGAGTTC	Intron 2, splice D2/A2.2
R2210	CGCCTGGAACACTGAAACCC	R2377	TCAACCCCAACTAACAGTTC	Intron 2, unspliced, pAp+pAd cleaved
R3180	TGGGTTTCAAGCACAAGTAG	R3342	TGCACCAGTGCTGGCTTCTG	Intron 2, unspliced, pAd cleaved
R4899	ACACCACAGGCATGGATACG	R5014	TGGGCGTTTAGTTACGCATC	Distal exon, pAd cleaved

Sequence of primers is derived from reference genome sequence NC_000883, as complementary either to a contiguous sequence or to a non-contiguous sequence. In primers marked with *, bases on the 5’ end are complementary to bases on the external side of a splice site, whereas bases on the 3’ end shown in italics are complementary to bases on the internal side of a splice site. RNA target is defined with respect to the functional map of B19 virus genome (Fig 1).

Real-time PCR and RT-PCR were carried out by using the RotorGene 3000 system (Corbett Research) and SybrGreen detection of amplification products. Amplification reactions were performed by using QuantiTect PCR SybrGreen PCR Kit (Qiagen) or QuantiTect SybrGreen RT-PCR Kit (Qiagen), including 10 pmol of each specific primer pair. For PCR, thermal profile consisted in 15 min at 95°C, then 40 cycles of 15 sec at 95°C, 30 sec at 55°C, and 30 sec at 70°C coupled with signal acquisition. For RT-PCR, two parallel reactions were performed for each sample, either including (RT+) or omitting (RT-) the reverse transcriptase from the reaction mix, and performing an initial step consisting in 30 min at 55°C, before the amplification reaction with a standard thermal profile. A final melting curve was performed, with thermal profile ramping from 50°C to 95°C at a 12°C/min rate, coupled with continuous signal acquisition.

Fluorescence emission recorded in the FAM/Sybr channel of the instrument was analyzed by using the functions available in the RotorGene 6.0 software. All reported experiments were carried out in duplicate series, and melting curve and quantitative analysis performed. Melting curve analysis was used for the determination of the specificity of the amplification products by defining, for each reaction, the melting profile and the Tm of the products.

For control and normalization with respect to the number of cells, a target sequence in the region of genomic DNA coding for 18S rRNA (rDNA) was selected and amplified by using the primer pair 18Sfor/18Srev. For quantitative evaluation of viral targets, the pair R2210-R2355, located in the central exon of B19V genome, was used for amplification of both viral DNA and total viral RNA, and absolute quantitation of viral DNA and total viral RNA was obtained by calibration to the standard DNA and RNA targets. For RNA targets, values for both RT+ and RT- reactions were obtained, and interference by amplification of residual DNA was evaluated by subtracting the residual RT- from the RT+ values. For the determination of the relative abundance of the different species within the whole set of viral transcripts, an array of primer pair combinations was used in group sets as indicated in Table 1. Relative quantitation of the different subsets of viral transcripts was obtained by efficiency-corrected comparative quantitation using LinRegPCR software [22], and subsequent normalization within each combination set and to the amount of total viral RNA. Data analysis was carried out by using the program GraphPad Prism version 5.00 for Windows (GraphPad Software, San Diego California, USA).

## Results

### EPCs dynamics and differentiation

**Fig 2:**
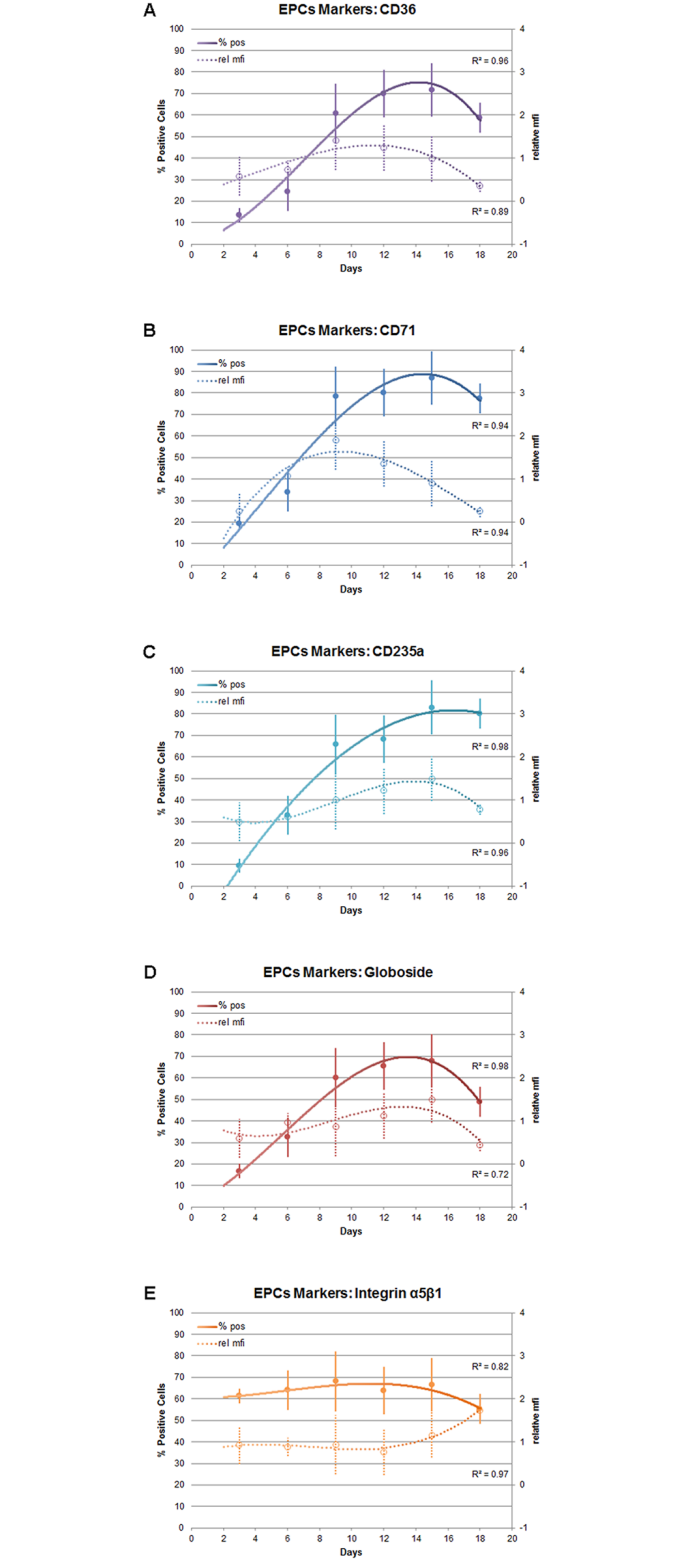
Phenotypic characteristics of differentiating EPCs. The PBMC-derived cell population was evaluated by cytofluorimetric analysis for the presence and abundance of erythroid differentiation markers (A: CD36; B: CD71; C: CD235a) and B19V receptor moieties (D: Globoside; E: α5β1 integrin). Each graph reports, at three days intervals, the percentage of cells positive for each indicated marker and the relative Mean Fluorescence Intensity (rel mfi), calculated as the mean geometric fluorescence for each sample, normalized to the average value within the fraction of positive cells. Reported curves are 3^rd^ order polynomial nonlinear fits, obtained from n total independent experimental data in the range 2–18 days of culture: for CD36, n = 49; for CD71, n = 35; for CD235a, n = 20; for Globoside, n = 32; for α5β1 integrin, n = 21. Source data are shown in S1 Dataset.

PBMC obtained from normal donors were grown in culture medium containing erythropoietic growth and differentiation factors. In these conditions, the cell population expanded and maintained viability for periods of time that normally extended up to 15–20 days. Within this period, the composition of cell population, as evaluated in replicate experimental series by cytofluorimetric analysis (Fig 2), changed towards a progressively higher proportion of cells showing the erythroid lineage-specific markers CD36, CD 71 and CD 235a, reaching highest values between days 9–15. Concerning B19V receptor moieties, the distribution of cells characterized by the presence of globoside was concordant with that of erythroid markers, opposed to the distribution of α5β1 integrin that was constant. Although a relatively high biological variability was observed in this process, these results indicated a progressive in vitro differentiation of an erythroid progenitor cell population susceptible to B19V infection.

### Viral replication in EPCs

**Fig 3:**
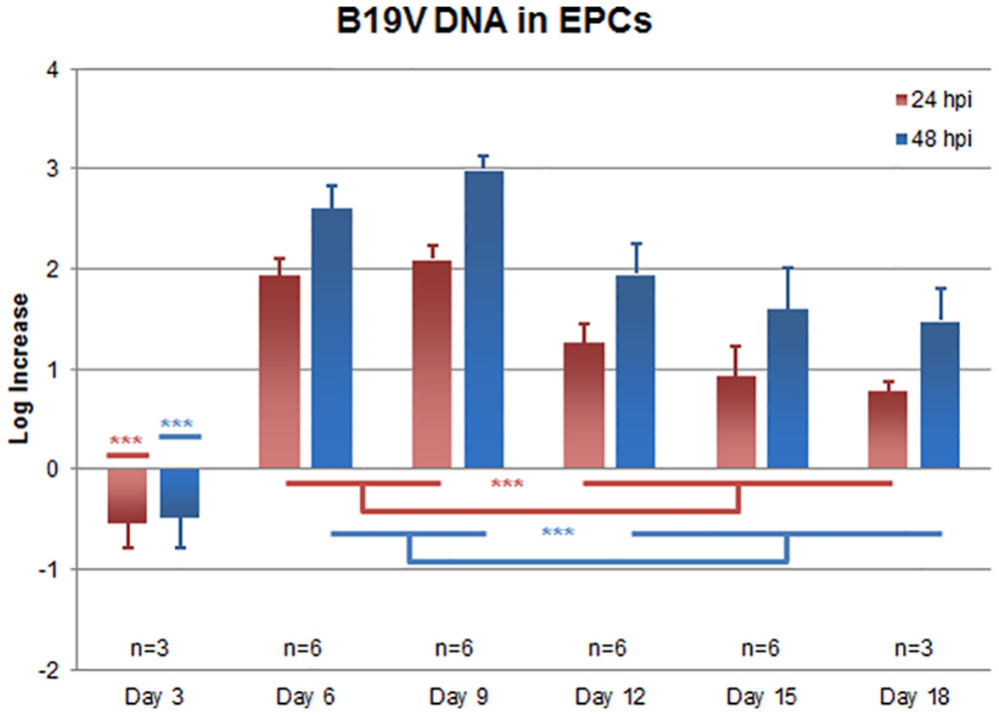
B19V replication in EPCs. EPCs were infected at different days from isolation with B19V, at the multiplicity of 10^3^ geq/cell. The amount of B19V DNA was determined by qPCR, and the Log increase in viral genome copies measured between 2 hours post-infection (hpi) and 24 or 48 hpi. Columns indicate the mean values obtained from independent experiments (n = 3–6), bars indicate the standard error of means. By one-way analysis of variance, statistical significance was obtained for mean values in both the 24 and 48 hpi series (p<0.001). By Tukey’s multiple comparison test, the following groups were significantly different (***, p<0.001): day 3 vs. days 6–9 and vs. days 12–18; days 6–9 vs. days 12–18. Source data are shown in S1 Dataset.

To determine the extent of viral replication in dependence on the degree of erythroid differentiation, EPCs at different days of in vitro culture, at 3 days intervals from 3 to 18 days post isolation, were infected with B19V for a 48 hours course of infection. Equal amounts of cells were collected at 2 hpi, 24 hpi and 48 hpi and the respective amounts of viral DNA were determined by qPCR (Fig 3). For the different days of culture and each infection course, the Log B19V DNA increase at 24 and 48 hpi versus 2 hpi was calculated as the mean of 3 to 6 independent experiments. At 3 days, cells were susceptible, but not permissive to B19V replication, showing a mean 0.5 Log decrease in the amount of viral DNA associated with cells. From day 6 to day 18 cells were susceptible and permissive to viral replication to different extents. Viral replication was maximal at days 6–9, with a mean 2.6–3.0 Log increase, while at days 12–18 cells were permissive to replication to a reduced degree, with a mean 1.5–2.0 Log increase. Overall, these results are evidence of the dependence of the viral replication efficiency on the differentiation stage of erythroid cells in the PBMC-derived cell culture system.

### Viral distribution in EPCs

**Fig 4:**
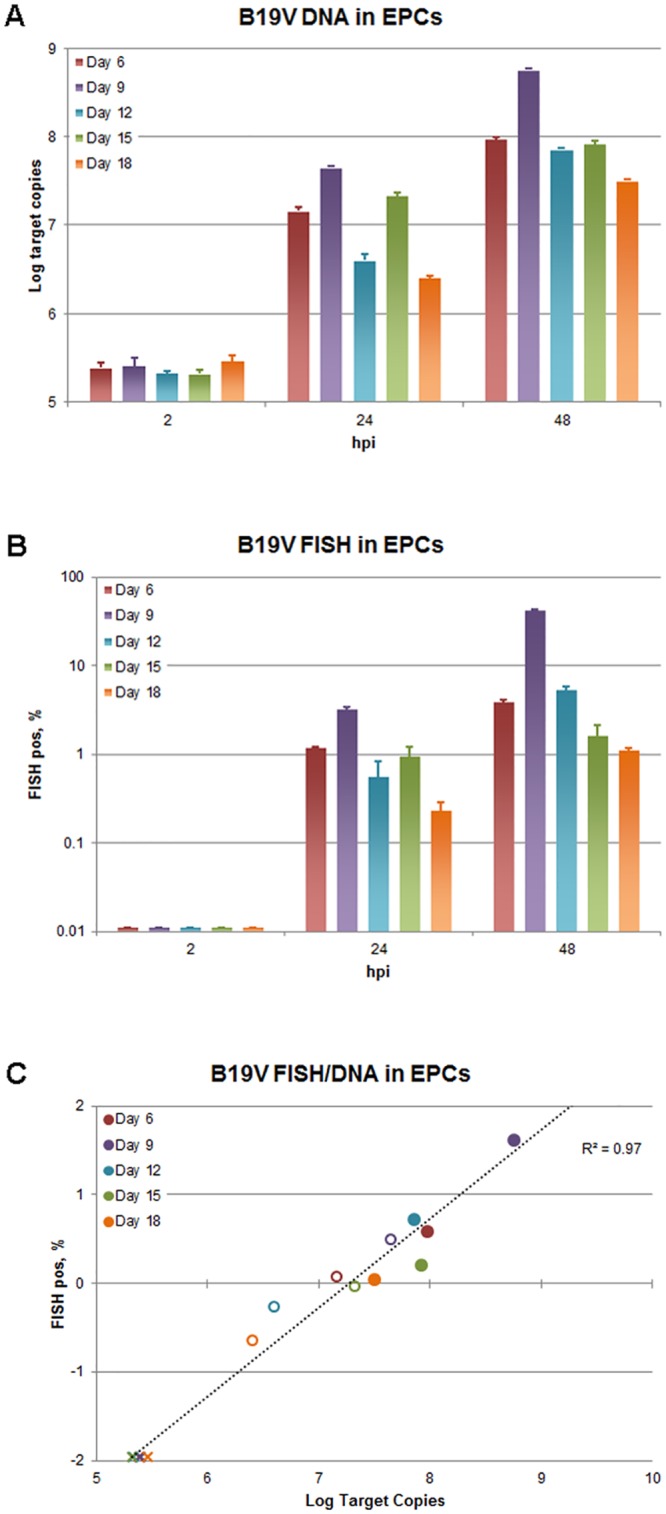
B19V replication and distribution in EPCs. EPCs were infected at different days from isolation with B19V, at the multiplicity of 10^3^ geq/cell, and analyzed at 2, 24 and 48 hpi by qPCR and flow-FISH. (A) Amount of B19V DNA determined by qPCR, Log DNA geq/10^4^ cells. (B) Fraction of positive cells determined by the flow-FISH assay. (C) Correlation of data obtained by the two different assays. X: samples at 2 hpi; ◯: samples at 24 hpi; ●: samples at 48 hpi; --- : linear regression fit. Source data are shown in S1 Dataset.

**Fig 5:**
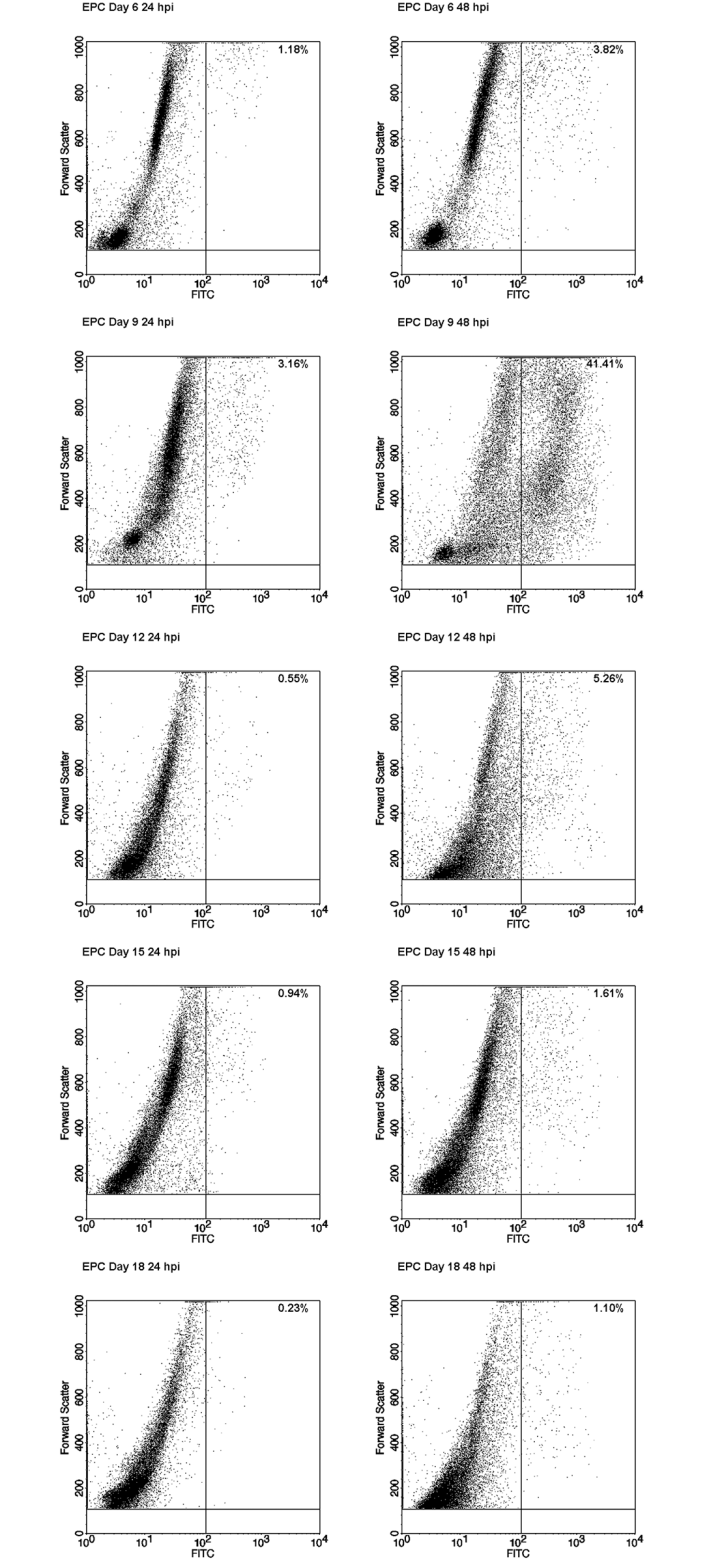
B19V replication and distribution in EPCs. The panel reports the dot plot graphs of flow-FISH assay in EPCs at different days of differentiation, at 24 and 48 hpi. The fraction of B19V positive cells (upper right quadrant) was evaluated by determining the number of cells above a fixed threshold along the X-axis (FITC) in the infected cell samples and subtracting the number of cells above threshold in respective uninfected negative controls.

Viral replication and distribution of productively infected cells within a differentiating population of EPCs was investigated in a selected representative experiment by parallel qPCR and flow-FISH assay. EPCs at different days of in vitro culture (6–18 days) were infected with B19V, and equal amounts of cells were collected at 2, 24 and 48 hpi. For each sample, qPCR analysis was carried out to determine the amount of viral DNA (Fig 4A), and flow-FISH assay was performed to determine the percentage of B19V positive cells (Fig 4B). In this experimental series, a steady increase in viral replication was observed for all sample series up to 48 hpi, with maximal replication at day 9 of differentiation. The observed increase in the amount of viral DNA was 3.4 Log for cells at days 9, 2.5–2.6 for cells at days 6, 12, 15, and 2.0 Log for cells at day 18. In accordance with these values, the percentage of FISH positive cells was higher (41.4%) at day 9, lower at days 6 (3.8%) and 12 (5.2%), and lowest at days 15 (1.6%) and 18 (1.1%). The dot-plot graphs (Fig 5) showed a diffuse pattern of positive cells, suggesting a continuous distribution in the amount of intracellular viral nucleic acids within the fraction of FISH positive cells. The mean amount of viral genome copies per cell could be calculated in the range 1.5–4.5x10^4^ for the 24 hpi samples, and 2.7x10^4^-1.0x10^5^ for the 48 hpi samples. The correlation of viral DNA amount and percentage of FISH-positive cells (Fig 4C) indicated that B19V replication was confined to a permissive subset of the potentially susceptible cells within the EPC population, not uniquely identified by any of the cell surface markers analyzed, but rather depending on the presence of other factors linked to the differentiation stages. Therefore, the extent of replication of virus in differentiating EPCs was mainly the result of the extent of recruitment of permissive cells within a heterogeneous population of potentially susceptible cells.

### Viral replication and expression in a time course of infection

**Fig 6:**
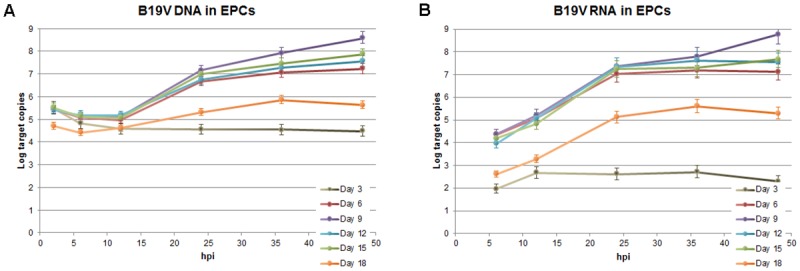
B19V replication and expression in a time course of infection in EPCs. Cells infected at different days from isolation at a moi of 10^3^ geq/cell were collected at the indicate time points post infection (2–48 hpi) and the amounts of viral nucleic acids determined by qPCR and qRT-PCR, for the different samples series. (A) Amount of B19V DNA determined by qPCR, Log DNA geq/10^4^ cells. (B) Amount of B19V total RNA determined by qRT-PCR, Log RNA target copies/10^4^ cells. Source data are shown in S1 Dataset.

A thorough systematic analysis of the dynamics of viral macromolecular synthesis in a time course of infection within EPCs at different days of growth and differentiations was carried out in a selected representative experiment. Cells at different days from isolation were infected with B19V, and equal amounts of cells were serially collected at early (2, 6, 12 hpi) and late (24, 36, 48 hpi) time points in a course of infection. The amount of viral DNA and total RNA were determined by qPCR and qRT-PCR, respectively, by using a same primer pair located in the central common exon of B19V genome, and normalized as Log target copies per 10^4^ cells (Fig 6).

For viral DNA (Fig 6A), the variation observed confirmed its dependence on the differentiation stage of erythroid cells. At 2 hpi, comparable amounts of viral DNA associated to cells were measured in cells from day 3 to 15 of culture, while lower amounts (-0.6 Log) were observed only for day 18. On the contrary, the extent of viral replication showed a correlation with the differentiation stage of EPCs. In cells infected at day 3, B19V was not able to replicate although viral DNA was detected during the whole time course (-1.0 Log from 2 to 48 hpi). In the other series, the increase within each course of infection confirmed the presence of a permissiveness window for viral replication. Between days 6 and 15, after a lag period (-0.2–0.5 Log from 2 to 12 hpi), viral replication started from 12 hpi with the highest increase in DNA amount occurring within 24 hpi (+1.6–2.0 Log from 12 to 24 hpi), and further increase up to 48 hpi (+0.6–1.4 Log from 24 to 48 hpi). Maximal B19V DNA replication was observed at day 9 (+3.1 Log from 2 to 48 hpi). At day 18, only low levels of replication were observed (+0.9 Log from 2 to 48 hpi).

For viral RNA (Fig 6B), the variation observed indicated a close association of genome replication and transcription. Only a very low level of transcription was observed for the day 3 series, while significant activity was detected for the days 6–15 series, and a lower but definite activity for the day 18 series. In detail, viral RNA was first detected at 6 hpi for all series, however, the amount of RNA was -2.3 Log lower for the day 3 series compared to the day 6–15 series, -1.6 Log lower for the day 18 series. Then, viral RNA increased similarly for all series until 12 hpi (+0.7–1.1 Log). Starting from 12 hpi, with the exception of the day 3 series where no further increment occurred, the amount of viral RNA increased in all other series in close association to the start of viral DNA synthesis, strongly until 24 hpi (+1.8–2.4 Log), marginally up to 48 hpi (+0.1–0.4 Log, with the exception of day 9 with a further +1.4 Log). These observations may be accounted for by a biphasic model, with early transcription occurring on the parental template, followed by late transcription on the replicating templates.

**Fig 7:**
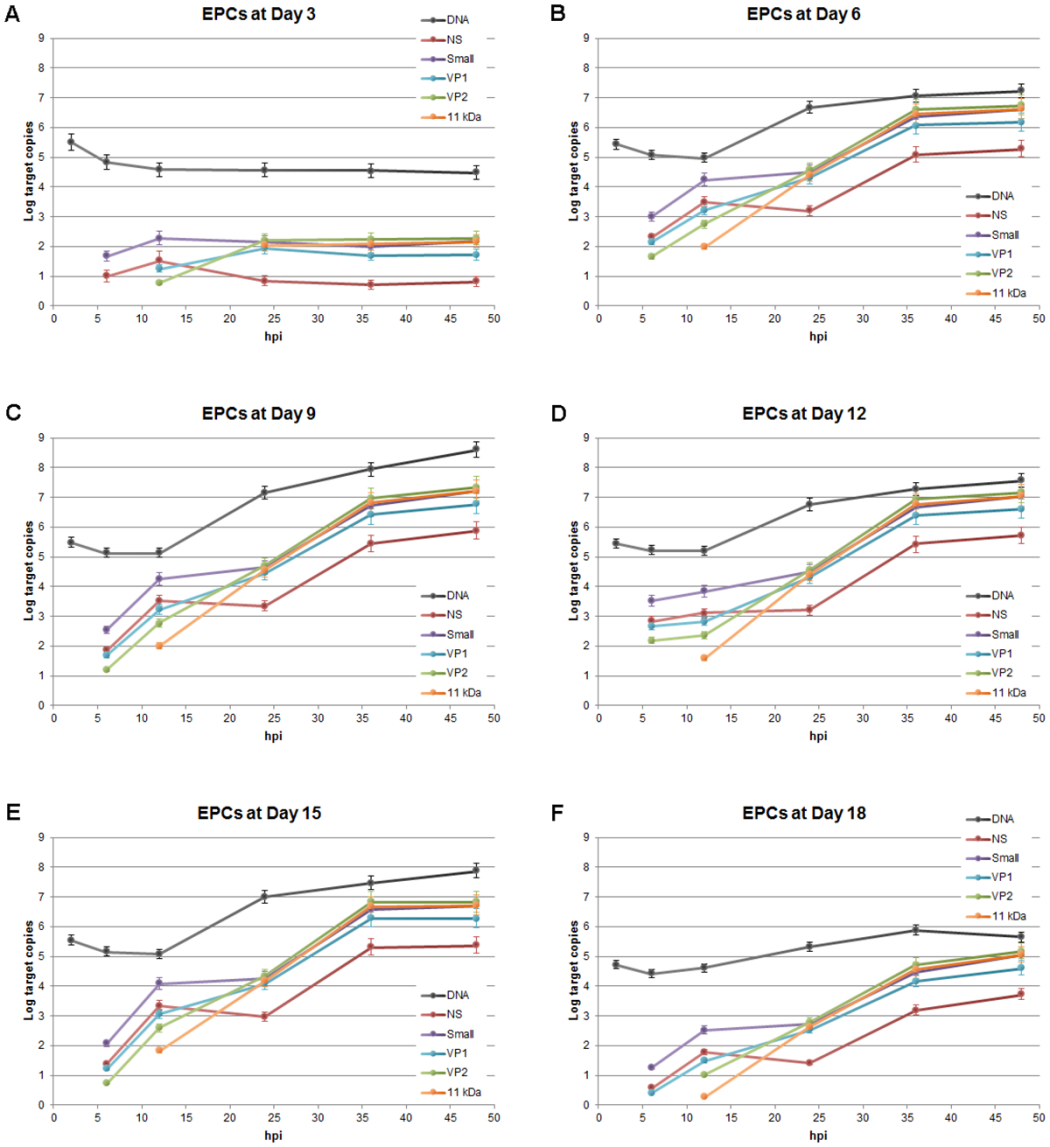
B19V replication and expression in a time course of infection in EPCs. The dynamics of accumulation of viral DNA and of the major classes of viral mRNAs in infected EPCs is reported for each sample series at different days from isolation, and for the different time points within the course of infection. The amount of the different classes of viral mRNAs was determined by qRT-PCR, using the selected primer pair combinations shown in Table 1, followed by normalization within each combination set and to the amount of total viral RNA. Source data are shown in S1 Dataset.

The set of mature viral mRNAs originates from pre-mRNA, due to the combination of splicing (from two introns, each with a donor and two alternative acceptor sites) and cleavage-polyadenylation events (presence of alternative sites in the center and right end of the genome) (Fig 1). A detailed analysis of the relative abundance of the different mRNAs classes was carried out by using a set of selective primers for qRT-PCR analysis (Fig 7). With the exception of the day 3 sample series, where the prominent feature was the low level of mRNA transcription, the dynamics of accumulation of the different classes of viral mRNA in the days 6–18 sample series was regular and largely independent on the degree of cellular differentiation. All classes of viral mRNAs continued to accumulate throughout the time course of infection, however, coherent with a biphasic model, an early pattern of expression at 6–12 hpi was replaced by a later pattern, from 24 to 48 hpi.

**Fig 8:**
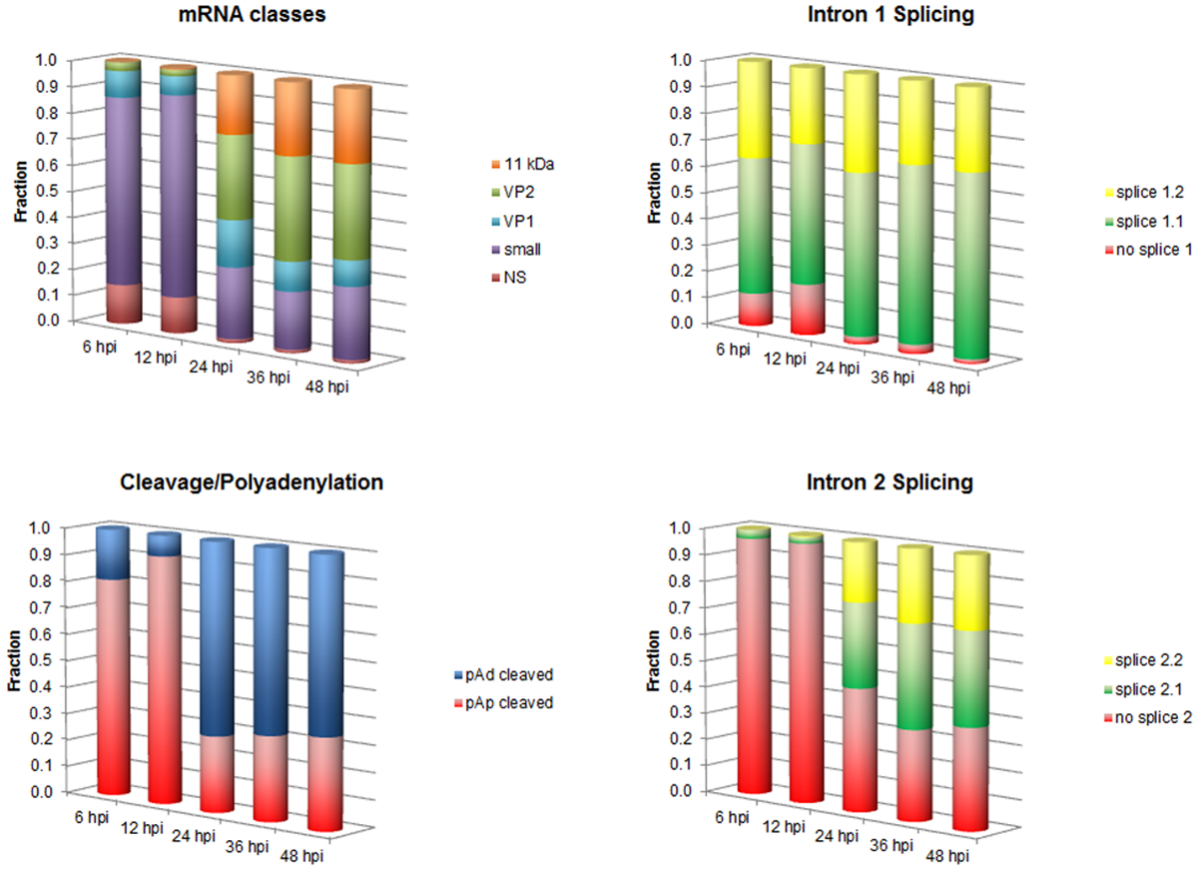
Transcript processing in a time course of infection in EPCs. Composite columns indicate the frequency of processing events at intron 1, intron 2 or pAp/pAd sites, and the resulting overall composition of the major classes of viral mRNAs. Cumulative data are averaged for each time point in the course of infection, for the days 6–18 experimental series. Source data are shown in S1 Dataset.

Early and late patterns of expression arose as a result of a changing frequency of mRNA processing events (Fig 8). In the early phase of infection, cleavage at the pAp site occurred at a frequency of 81–92%, while in later phases of infection cleavage at the pAd site accounted for 66–72% of events. In the processing events of intron 1, 12–19% in early phases, but only 0.2–0.3% in late phases did not undergo splicing, while in the remaining cases splicing occurred 1.4–2.2 times more frequently at acceptor site A1-1 over A1-2, increasingly at later times post-infection. In the processing events of intron 2, 97% in early phases and 34–46% in late phases did not undergo splicing, while in the remaining cases splicing occurred only at acceptor site A2-1 in early phases, and 1.3–1.4 times more frequently at acceptor site A2-1 over A2-2, at late times post-infection. As a consequence, the relative composition of viral mRNAs classes clearly distinguished an early from a late pattern of expression profile. In the early phase, before the onset of viral replication, mRNAs were mostly cleaved at the proximal site, leading to a higher relative abundance of small mRNAs and mRNA coding for NS protein. In the later phase, in coincidence with viral DNA replication, mRNAs were mostly cleaved at the distal site, leading to a higher relative abundance of mRNAs for VP1/2 and 11kDA proteins. The overall finding was therefore a confirmation of the linked coordination of viral DNA replication, viral RNA transcription, and differential usage of mRNAs processing signals to achieve a productive replicative cycle in EPCs.

### Virus release and infectivity

**Fig 9:**
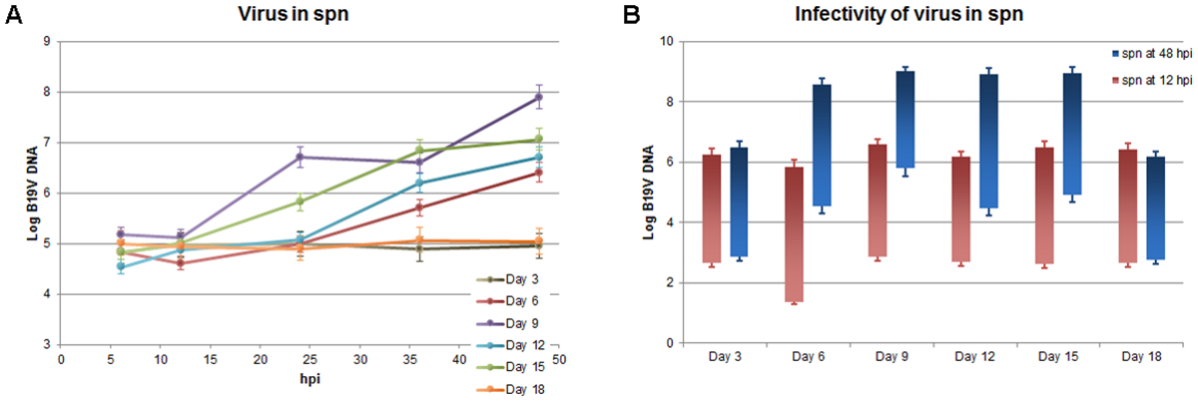
Virus release from infected EPCs and infectivity. (A) Variation of viral DNA amounts released in the supernatants (spn) of B19V-infected EPCs, at different time points post-infections for EPCs at different days from isolation. Log of viral DNA target copies (Log DNA geq/100 μL) is reported for the different samples series. (B) Infectivity of virus released from EPCs. Supernatants recovered at 12 hpi and 48 hpi from B19V-infected EPCs at different days of culture (day 3–18) were used to infect a new population of EPCs at day 9 of culture, and the amount of intracellular viral DNA at 2 and 48 hpi determined by qPCR assay. DNA amounts are reported as variation bars: lower limits of bars are the amount of viral DNA detected at 2 hpi, upper limits are the amount detected at 48 hpi (Log DNA geq/10^4^ cells). By one-way analysis of variance, statistical significance was not present for the 12 hpi series, but achieved for the 48 hpi series (p<0.001). By Tukey’s multiple comparison test, the following groups within the 48 hpi series were significantly different (p<0.001): day 3 vs. days 6–15; days 6–15 vs. day 18. Source data are shown in S1 Dataset.

In the same experiment, the release of virus in the supernatant of infected cell cultures and its ability to start a new infectious cycle were determined (Fig 9). Viral DNA accumulated in the supernatant to extents proportional to what present inside cells for most samples (Fig 9A). In the day 3 series, a non-permissive environment, DNA remained to background values for the whole time course. In the day 6–15 series, viral DNA accumulated from 24–36 hpi up to 48 hpi (+1.9–2.8 Log increase over background values). An exception was constituted by the day 18 cell series, where, as opposed to a modest but defined increase in intracellular DNA, no increase was observed in the supernatant with respect to background values.

To assess infectivity of virus released from EPCs, volumes of 100 μL of supernatants obtained at 12 and 48 hpi from each samples series were used for a further cycle of infection of 10^6^ EPCs at day 9 of culture (Fig 9B). The amount of viral DNA within infected EPCs was then determined at 2 and 48 hpi, in order to evaluate a possible viral replicative activity. Supernatants obtained at 12 hpi for each of the tested sample series showed residual infectivity, leading to a mean increase of 3.8 Log in infected EPCs from 2 to 48 hpi. Supernatants obtained at 48 hpi yielded infectivity results coherent with the amounts of virus present in supernatants. Same amounts of virus and infectivity values as the 12 hpi supernatants were observed for the supernatants obtained for the days 3 and 18 samples series (mean increase 3.5 Log), while increased amounts of virus with similar infectivity were observed in supernatants for the sample series at days 6–15 (mean increase of 4.4 Log), indicating the production and release of infectious virus. These results confirm the restriction of a productive viral cycle to a limited differentiation phase in EPCs, also indicating the presence of a restriction step within cells at late phase of differentiation, affecting the maturation of virus and preventing the release of infectious virus from cells. On the other hand, these results highlight how differentiating EPCs can be a suitable cell system for the in vitro production of infectious virus.

## Discussion

Erythroid progenitor cells in bone marrow, or the equivalent during fetal development, are the main target cell population for B19V and constitute the central paradigm for the description of B19V lifecycle and characteristics of virus-cell interactions [23–29]. However, B19V can efficiently replicate also in erythroid progenitor cells obtained from diverse sources including peripheral blood [30–32], thus providing a readily available option for primary cells equally suitable for the study of B19V virus-cell interactions. Human CD34+ cells isolated from mobilized stem cells in peripheral blood can be cultured and differentiated in vitro toward a population of more mature erythroid cells able to support B19V replication [13,33]. Enrichment and selection are not necessary, so that the heterogeneous population of cells in the PBMC fraction can equally yield a population of growing and differentiating erythroid progenitor cells supporting B19V replication [14]. In our experimental conditions, following established protocols, PBMC-derived cells could grow and differentiate into a population of EPCs that showed increasing presence of erythroid specific markers, such as CD36, CD71 and CD235a. In the frame of this differentiation wave of erythroid progenitor cells, the capacity of cells to support B19V replication, and the specific pattern of intracellular events leading to a productive replicative cycle, followed a dynamics highly related to the differentiating stage of erythroid progenitor cells.

Tropism of B19V for EPCs is mainly determined by the presence on cell surface of the principal receptor, globoside [34–36]. The distribution of globoside within differentiating EPCs follows closely that of other erythroid markers, indicating that this cell population on the whole is potentially susceptible to B19V infection. Other cellular moieties on EPCs have been involved in B19V attachment and penetration. A VP1u binding molecule, recently reported to be essential for B19V, shows a frequency distribution on EPCs closely following their differentiation [37,38], while α5β1 integrins, also reported as essential for B19V [39,40], are evenly distributed and independent of the differentiation stage.

The set of erythroid differentiation markers analyzed, as well as the same B19V receptor moieties, are not accurate classifiers of the cells supporting B19V replication. The extent of viral replication, determined by qPCR, as well as the fraction of productively infected cells, determined by flow-FISH assay, indicate that cells can support B19V more at earlier differentiation stages, between 6–9 days, compared to later stages of erythroid differentiation occurring at 12–15 days [10–12]. Furthermore, even at the relatively high moi used in our studies, the extent of viral replication is correlated to the fraction of productively infected cells, that is constantly lower than that showing the presence of erythroid markers and receptor moieties.

The presence and distribution of viral receptors within EPCs will define the set of susceptible cells, but the effective outcome of infection will therefore depend on a series of intracellular events, conditioned by the cell differentiation stage and specifically related to viral macromolecular synthesis. A detailed analysis of viral macromolecular synthesis, obtained by qPCR and qRT-PCR, clearly indicates the occurrence of specific patterns related to the specific differentiation stages of EPCs and leading to diverse outcomes of infection. In particular, early-stage progenitors are characterized by an abortive infection, opposed to later progenitors that can support a productive infection, characterized by a biphasic profile of macromolecular synthesis.

In EPCs at a very early stage (3 days), the abortive pattern of infection is characterized by absence of genome replication and minimal transcriptional activity. This pattern is similar to what detected in other cell types or several cell lines, that are considered susceptible but not permissive to B19V. Restriction may affect the efficiency of the initial phase of B19V lifecycle, such as the penetration, uncoating, and intracellular localization steps, as well as DNA second-strand synthesis, in this case preventing the generation of a functionally active genomic template.

In EPCs at later stages (6–18 days), the productive pattern of infection is constituted by biphasic, coordinated replication and transcription events. Assuming the occurrence of second strand synthesis and generation of a functionally active genomic template, an early phase characterized by transcription on the parental genome is followed by a late phase characterized by both replication and transcription on the amplifying genome. mRNA processing events can also be differentiated on this basis, as in the early phase cleavage at pAp site is prevalent, splicing is relatively low, and mRNA for NS protein is produced in relatively higher abundance, while in the late phase cleavage at pAd site is prevalent, splicing is relatively high, and mRNAs for the VP and the 11 kDa proteins are produced in abundance.

This observed dynamics of macromolecular synthesis is in agreement and confirms what observed in other experimental systems, including both bone marrow cells and cell lines such as UT7/EpoS1 [20,21,41]. A series of cis-acting sequences have been mapped on B19V genome and their role in DNA replication and RNA processing experimentally determined [42–44]. The viral NS protein plays a key role in regulating viral macromolecular synthesis [44,45], however our results indicate the necessary involvement of erythroid-, stage-specific intracellular factors, possibly acting in partnership with NS protein, functional to the regulation of viral macromolecular synthesis. The presence of such factors would constitute an effective molecular switch, able to consent the transition between the early and late viral replicative phases and achievement of a productive replicative cycle in a subset of erythroid progenitor cells. Finally, this productive pattern of macromolecular synthesis can lead to release of virus from infected EPCs, but at a very late stage (18 days) an impaired release of virus indicates the presence of further levels of restriction affecting the late phases of the viral lifecycle.

**Fig 10:**
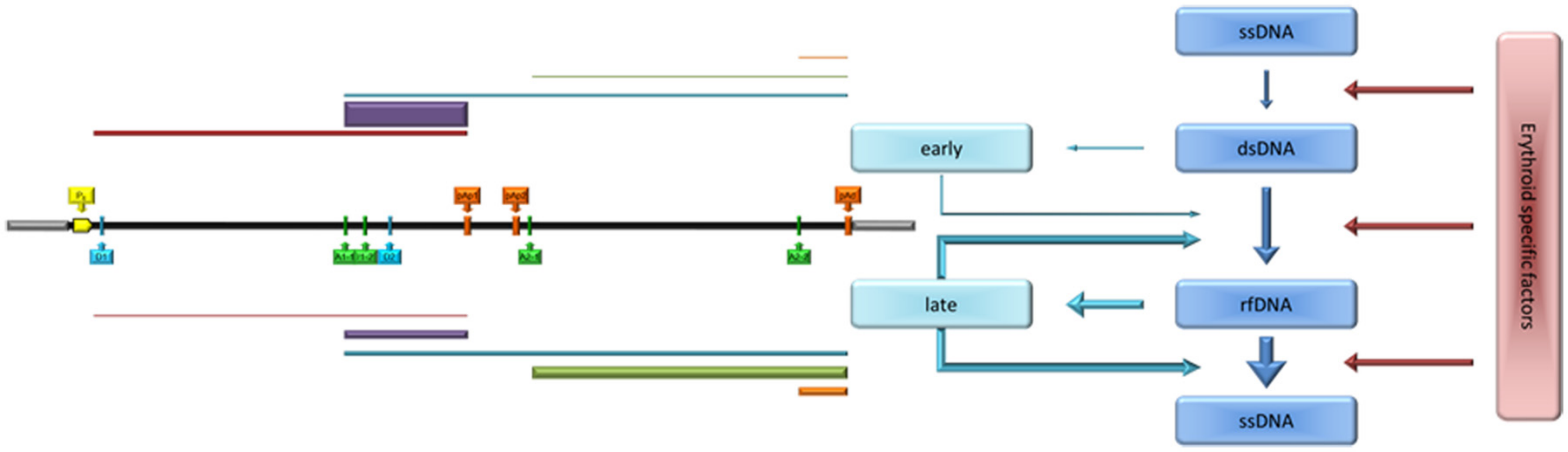
A model for B19V replication and expression. Viral genome can be present in four consecutive states, connected by three state transitions: input ssDNA, initial dsDNA, replicating rfDNA, and product ssDNA. Two functional profiles are identified as “early” (from dsDNA) and “late” (from rfDNA), characterized by a differential abundance and relative composition of transcriptome (compare map in Fig 1). Each profile is involved in regulative loops on genome state transitions. Erythroid specific factors are critical in regulating state transitions and are dependent on the differentiation and physiological state of the cell.

The outcome of infection will depend on the capacity of the virus to proceed through a series of steps, in dependence of cellular factors (Fig 10). Comparative analysis of the erythroid- and differentiation-specific expression profiles may define the range of intracellular factors determining a permissive or restrictive environment for B19V, however considering that several mechanisms and intracellular pathways can be involved [46,47]. B19V interacts with the cellular DNA Damage Response [48–50], modulates progression through cell cycle [51–53], cell differentiation [54] and induction of apoptosis [55–58]. B19V depends for its replication on Epo stimulation [26,27] and Epo receptor activation [59], and viral replication is enhanced by hypoxic environment through a signal activation cascade [60,61]. Innate immune response mechanisms may also be relevant to determine the outcome of infection [62]. In turn, the diverse replicative patterns and productive outcomes, in dependence of the cell differentiation stages, might lead to different consequences on the cell population dynamics and cell conditions (e.g., cell apoptosis and/or cell death), a working hypothesis that can be matter of subsequent investigation.

Overall, these results highlight the very tight adaptation of B19V to a specific cellular target defined both by its erythroid lineage and its differentiation stage. The evolutionary success of the virus is strikingly linked to its ability to infect a labile, self-renewing cell population without affecting neither the more undifferentiated cells with staminal properties, nor the terminally differentiated cells with scarce potential to support replication. While earlier studies indicated that B19V could infect bone-marrow derived erythroid progenitor cells at the BFU-E and CFU-E stage, increasingly as cell differentiation increased [63], in the PBMC-derived EPCs system the more undifferentiated cells corresponding to the CFU-E stage are non-permissive, while the permissive cell population can better be identified as cells at the ensuing proerythroblast stage [13,14].

As a final observation, our study presents evidence of a substantial production of infectious virus obtained in an in vitro system, constituted by primary cells alike the natural target cells infected in vivo. Not least important, the in vitro generated EPCs, with their full permissiveness, ability to support virus replication, and ability to yield infectious virus, are a valid system in the search and evaluation of compounds with a specific antiviral activity against B19V [64].

## Supporting Information

S1 Dataset Dataset for Figs 2, 3, 4, 6, 7, 8, 9. (XLSX)
